# Medial-to-Lateral Approach to the Splenic Flexure Resection and End Transverse Colostomy: A Case Report and Operative Video

**DOI:** 10.7759/cureus.30442

**Published:** 2022-10-18

**Authors:** Lorna Kang, Truong Ma

**Affiliations:** 1 General Surgery, Summa Health, Akron, USA; 2 Colon and Rectal Surgery, Summa Health, Akron, USA

**Keywords:** minimally invasive, obstruction, colostomy, colon cancer, laparoscopy, splenic flexure

## Abstract

Splenic flexure cancer is relatively rare among colon cancers. We present a case of a 76-year-old female with a partially obstructing tumor in the proximal descending colon and pulmonary metastasis who underwent a laparoscopic resection of the splenic flexure with end transverse colostomy due to clinical symptoms of obstruction. This case highlights several important technical considerations in safely performing the laparoscopic resection of the splenic flexure through the medial-to-lateral approach starting at the plane below the inferior mesenteric vein (IMV), taking care to preserve the tail of the pancreas. We present a narrated video demonstrating our approach, taking care to highlight important anatomic landmarks.

## Introduction

Splenic flexure cancer is relatively rare compared to adenocarcinoma located in the descending or sigmoid colon, representing 1%-8% of all colon cancers [1]. Tumors located in this region often present late in advanced stages, most commonly with obstructive symptoms [2]. Currently there are no standardized techniques for splenic flexure resection; segmental resection, extended right or left hemicolectomy have been described. Laparoscopic splenic flexure mass resections are technically challenging due to the risk of injury to the spleen and pancreas, as well as the presence of various feeding branches from the inferior and superior mesenteric vessels [3]. The identification of the middle and left colic arteries and the construction of the anastomosis can also be challenging in the laparoscopic approach.

We present a case of a 76-year-old female with a partially obstructing tumor in the proximal descending colon and pulmonary metastasis who underwent a laparoscopic resection of the splenic flexure with end transverse colostomy due to clinical symptoms of obstruction. The patient tolerated the procedure well and there were no perioperative complications. Through the case presentation and operative video, we highlight several important technical considerations in safely performing the laparoscopic resection of the splenic flexure through the medial-to-lateral approach starting at the plane below the inferior mesenteric vein (IMV), with a special focus on identifying and dissecting around the tail of the pancreas. Informed consent was obtained from the patient for video recording for the purposes of medical education.

## Case presentation

Case description

The patient is a 76-year-old female who presented to the colorectal surgery clinic with left-sided abdominal pain, and occasional nausea without emesis. She had a CT of the abdomen and pelvis which demonstrated a partially obstructing splenic flexure mass as well as bilateral pulmonary nodules likely representing metastatic lesions. Diagnostic colonoscopy showed a circumferential partially obstructing tumor in the proximal descending colon which was tattooed and biopsied. One week following the colonoscopy, she was admitted to the hospital with large bowel obstruction. The decision was made to proceed with laparoscopic splenic flexure resection and end transverse colostomy due to symptoms of complete large bowel obstruction. The medial dissection began with the identification of the IMV, en bloc resection of the Gerota’s fascia and anterolateral abdominal wall associated with the splenic flexure mass, followed by lateral mobilization, vascular ligation, and division of the colon (Video 1). A transverse colostomy was created following the laparoscopic portion of the case. The patient tolerated the procedure well and had an uneventful post-operative course. She was discharged home on post-operative day 9. 

**Video 1:** Laparoscopic medial-to-lateral approach to the splenic flexure resection.

Operative details

**Figure 1:**
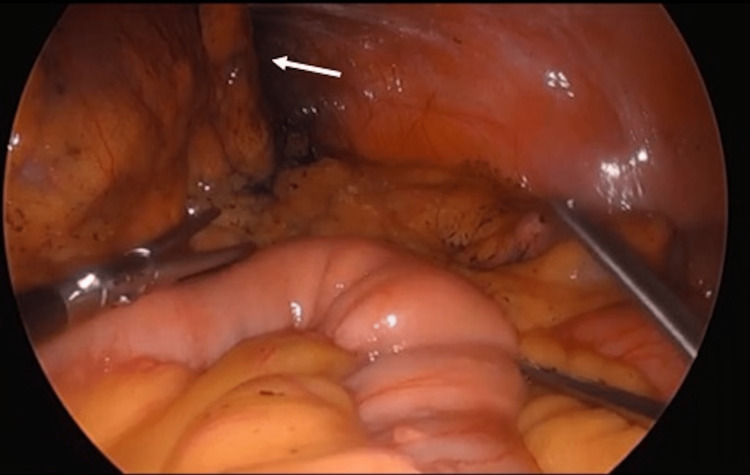
The tattooed mass is visualized in the left upper quadrant, adherent to the anterolateral abdominal wall (white arrow).

**Figure 2:**
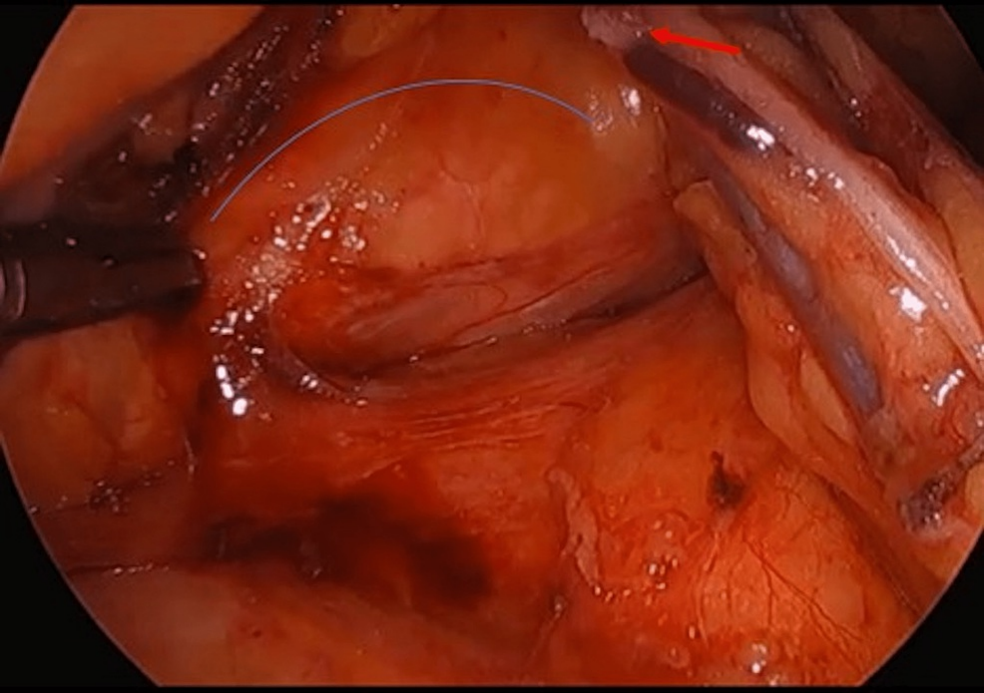
The dissection is carried out through the plane between the mesocolon and retroperitoneum at the anterior border of the pancreas (blue line). The IMV has been divided to facilitate this dissection (red arrow). IMV, inferior mesenteric vein

**Figure 3:**
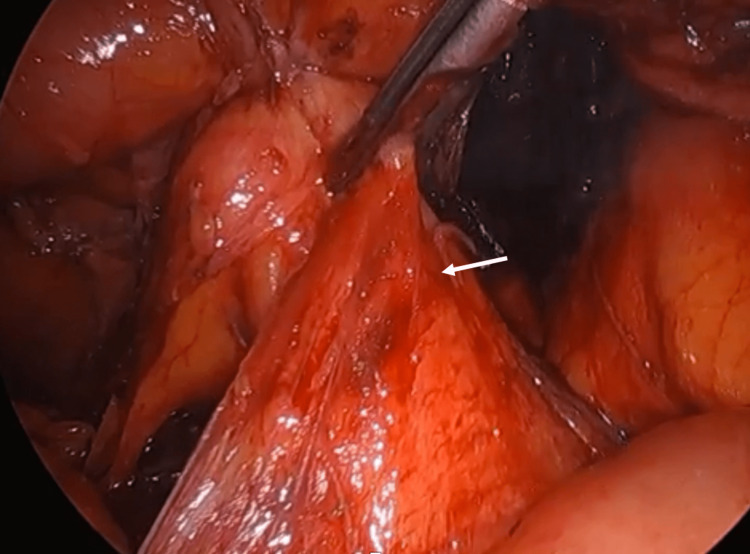
The posterior margin of the splenic flexure mass invades the Gerota's fascia (white arrow).

**Figure 4:**
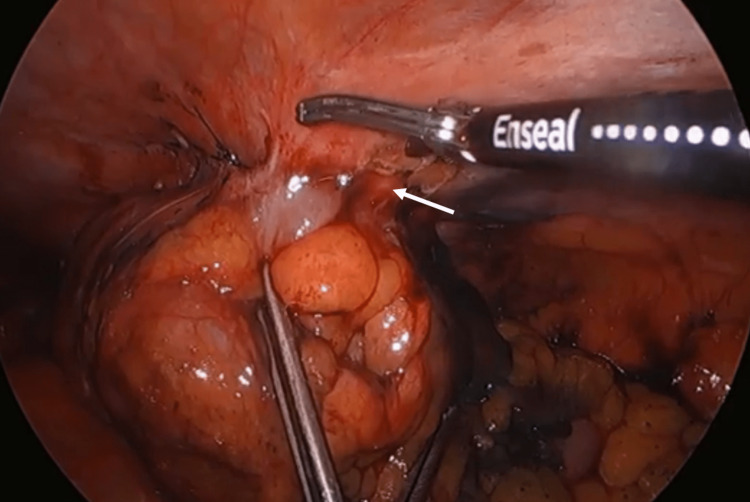
The anterior margin of the splenic flexure invades into the anterolateral abdominal wall (white arrow).

The patient received a subcutaneous heparin injection and preoperative antibiotics were administered within 30 min of the initial incision. The patient was positioned supine with arms tucked and legs placed in stirrups. A Veress needle was placed in the left upper quadrant and a 5 mm optical trocar was placed. Three additional trocars were placed under direct visualization; umbilical, right upper, and right lower lateral positions. We began with the survey of the abdomen which was negative for obvious metastatic lesions. The previously tattooed splenic flexure mass was found adherent to the anterolateral abdominal wall (Figure 1). Due to the difficult location and bulky nature of the mass, we chose the medial-to-lateral approach. We identified the ligament of Treitz, divided the IMV, and started our medial dissection below the IMV towards the lateral sidewall, lifting the transverse mesocolon anterior to the tail of the pancreas and dissecting through the plane anterior to it in order to keep the pancreas in the retroperitoneal plane (Figure 2). We then carried out the lateral dissection along the line of Toldt along the proximal descending colon. The lesser sac was entered at the gastrocolic ligament and continued towards the splenic flexure, taking care to preserve the gastroepiploic artery. The mass at the splenic flexure invaded the Gerota's fascia posteriorly and the abdominal wall anterolaterally, requiring en-bloc resection of the invaded tissue (Figures 3-4). The remaining attachments at the splenic flexure were mobilized. We divided the left branch of the middle colic and left colic arteries, completed the mesentery division, and divided the colon at the transverse and sigmoid colon with the laparoscopic stapler, leaving a long Hartmann pouch. The specimen was removed through a Pfannenstiel incision. A colostomy site was created at the left upper quadrant, the proximal end of the colon was exteriorized through this incision, and an end transverse colostomy was fashioned. The operative time was 141 min.

The final specimen was 37.5 cm in length. The tumor was located 21 cm from the proximal margin and 17 cm from the distal margin. Pathological examination showed invasive moderately differentiated colorectal adenocarcinoma with negative margins, and invasion through the muscularis propria with inflammatory adhesions to pericolorectal tissues, with three out of 12 nodes positive. The pathologic stage was T3N1b.

## Discussion

The splenic flexure is located between the terminal portion of the midgut and the beginning of the hindgut. During the early stages of embryological development, the anterior sheet of the transverse mesocolon fuses to the posterior wall of the greater omentum and the transverse and descending mesocolon partially fuse together at the inferior pole of the spleen. The posterior sheet of the descending mesocolon then fuses with the left retroperitoneal wall. This forms the splenic flexure of the colon, located at the distal transverse and proximal descending colon [4]. The splenic flexure receives blood supply from the left branch of the middle colic artery supplied by the superior mesenteric artery and the left colic artery from the inferior mesenteric artery. The lymphatic drainage at the splenic flexure is primarily directed toward the left colic vessels [5].

Given the dual blood supply to the splenic flexure of the colon and its location at the junction of the hindgut and midgut, lymphadenectomy for cancer can be challenging given the complex vascular supply in this region of the colon. There is no established surgical approach to splenic flexure cancers given its rarity, some surgeons advocate for extended hemicolectomy over segmental resection not only for adequate lymph node harvest but also to preserve blood supply to the anastomosis in cases where intestinal continuity is restored. El-Hendawy et al. compared segmental resection, extended left, or left hemicolectomy for splenic flexure cancer and reported no difference in oncologic outcomes among the three approaches [6].

## Conclusions

We concluded that laparoscopic splenic flexure resection can safely be performed by taking meticulous care to identify major vascular structures and retroperitoneal structures such as the pancreas. For large bowel obstruction in the setting of an obstructing malignant mass, colon resection and fecal diversion can provide symptomatic relief.
